# Dietary Nitrate Protects Against Skin Flap Ischemia-Reperfusion Injury in Rats *via* Modulation of Antioxidative Action and Reduction of Inflammatory Responses

**DOI:** 10.3389/fphar.2019.01605

**Published:** 2020-01-22

**Authors:** Hao Cui, Yuanyong Feng, Chuanliang Shu, Rongtao Yuan, Lingxue Bu, Muyun Jia, Baoxing Pang

**Affiliations:** ^1^ Department of Oral and Maxillofacial Surgery, The Affiliated Hospital of Qingdao University, Qingdao University, Qingdao, China; ^2^ School of Stomatology of Qingdao University, Qingdao, China; ^3^ Department of Stomatology, The Affiliated Qingdao Hiser Hospital of Qingdao University, Qingdao, China; ^4^ Qingdao Municipal Hospital, Affiliated to Shandong University, Qingdao, China

**Keywords:** nitrate, ischemia-reperfusion injury, nitric oxide, oxidative stress, inflammation, apoptosis

## Abstract

Dietary nitrate, found abundant in green vegetables, can be absorbed into the blood and be converted to nitric oxide (NO) in the body. Dietary nitrate has been proved to have many positive physiological functions in the body. Here, we evaluated the therapeutic effects of dietary nitrate on skin flap recovery following ischemia reperfusion (IR). Wistar rats were pretreated with nitrate from one week prior to ischemia to the end of reperfusion. It was found that oral administration of nitrate increased serum nitrate and nitrite levels, protected cells from apoptosis, and attenuated flap tissue edema. In the meantime, the oxidative stress marker malondialdehyde was reduced, while the activities of antioxidant enzymes were restored after nitrate treatment. Moreover, the macrophage and neutrophil infiltration in the flap was significantly attenuated by nitrate supplementation, as were the pro-inflammatory cytokines. In sum, we found that oral administration of nitrate can attenuate skin flap IR injury through the regulation of oxidative stress and inflammatory response.

## Introduction

Skin flap transplantation is widely used in plastic surgery. Although surgical technologies have been improving continuously, flap failure caused by ischemia reperfusion (IR)-induced flap failure is still a tough clinical challenge (Bai et al., 2016). A long period of vascular exclusion may lead to skin flap loss if the process of IR injury is not effectively addressed (van den Heuvel et al., 2009). Ischemia tissue experiences vessel and capillary caliper, endothelial cell dysfunction, and inflammatory mediator production (Carroll and Esclamado, 2000). Thus, the reperfusion of ischemic tissue is an important kind of body repair mechanism. However, the restoration of blood flow to ischemic tissue triggers another cascade of pathophysiological events that results in tissue injury *via* promoting the production of oxygen radicals and the production of inflammatory factors.

IR injury in skin flap can be protected against to some extent by suitable preconditioning (Xiao et al., 2015). Many strategies aimed at preventing skin flap failure have been proposed. For example, hyperbaric oxygen preconditioning and ischemic preconditioning were demonstrated to provide a promising clinical result (Joo et al., 2006; He et al., 2011; Losada et al., 2014; Hausenloy and Yellon, 2016). However, the long-term effect and safety of clinical preconditioning still require further investigation. Many pharmacological therapies (e.g., melatonin, aprotinin, and heparin) have also been shown to improve flap tolerance (Wright et al., 1988; Gurlek et al., 2006; Polat et al., 2008), but few drugs are currently available for clinical application. Thus, it will be meaningful if a treatment strategy can prevent IR injury clinically.

Nitrate serves as a natural constituent of the human diet. Green leafy vegetables, including beetroot juice, spinach, and celery, are major contributors to nitrate (Lundberg et al., 2011b). Approximately 85% of the total dietary nitrate intake is derived from vegetables (Myint et al., 2007). There is increasing evidence to indicate that some beneficial effects of dietary nitrate, which may boost the nitrate-nitrite-NO pathway (Castiglione et al., 2012; Sindler et al., 2014a). Diets with nitrate supplementation have aroused interest since they can attenuate oxidative stress, reverse vascular dysfunction, increase mitochondrial efficiency, and enhance the skeletal muscle contractile properties of animals and humans (Carlstrom et al., 2011; Rammos et al., 2014; Hezel et al., 2016b; Zafeiridis et al., 2019). In addition, the salutary effects of nitrate preconditioning have been proposed in IR injury models of the heart and kidney (Sobierajski et al., 2013; Jeddi et al., 2016; Yang et al., 2017). Our previous research showed that dietary nitrate can increase ischemia skin flap survival through increased blood flow and reduction of the inflammatory response (Cui et al., 2019). However, at present, studies evaluating the potential of oral nitrate supplementation to attenuate skin flap ischemia-reperfusion injury are scarce.

Therefore, this study observed whether dietary nitrate supplementation has any effect on skin flap IR injury and then further protects the body from skin flap loss. It was showed for the first time that oral administration of nitrate can attenuate skin flap IR injury by triggering an increase in antioxidant capacity and a reduction in the inflammatory response.

## Materials and Methods

### Animals and Nitrate Supplement

Twenty-four male Wistar rats (220 ± 10g, 2 months old) were obtained from Pengyue Laboratory Animal Breeding Center (Jinan, China) and were housed under consistent conditions (temperature: 24 ± 3°C, humidity: 55 ± 5%, light/dark cycle: 12 h). All animal experiments were approved by the Committee of Animal Care and Welfare in the Affiliated Hospital of Qingdao University (Protocol No. AHQU20190107A). After 1 week of acclimation, rats were randomly divided into four groups: the sham (distilled water, without IR), IR (distilled water, IR), IR + NaCl (5 mmol/L NaCl), and IR + nitrate (5 mmol/L NaNO_3_) groups, with six rats in each group. Rats in the IR+nitrate group were orally administered sodium nitrate dissolved in distilled water (5 mmol/L) for 7 days before surgery. An equal concentration of sodium chloride served as the IR+NaCl group. The remaining rats were orally administered distilled water.

### Surgical Procedure for the IR Injury of the Flap Model

After anesthesia through ketamine (80 mg/kg) and xylazine (10 mg/kg) injections, rats were placed on a heating pad to maintain a constant body temperature throughout the surgery. After surgical cleansing of the dorsal area, a rectangular island flap (3 × 4 cm) was elevated on the dorsum, retaining only the deep circumflex iliac (DCI) vessels as the vascular pedicle (**Supplemental Figure 1**). This island flap contained skin, subcutaneous tissue, and panniculus carnosus muscle. The viability of the island flap was confirmed with a pilot study before launching the experiment. Ischemia was induced by placing microvascular clamps on the pedicles of the flap. In the IR, IR+NaCl, and IR+nitrate groups, the deep circumflex iliac artery of the rats was clamped with a microvascular clamp for 10 hours. At the end of ischemia induction, the flap was resutured with 3-0 silk suture. Reperfusion was initiated by removing the clamp and was confirmed by the reconstitution of the blood flow of the tissue. The ﬂaps in the sham group were free from ischemia induction and were resutured with 3-0 silk suture.

### Preparation of Specimens and Homogenization

After 12 h reperfusion, all of the rats were deeply anesthetized and killed humanely. Blood samples were collected, and the serum was prepared by centrifugation at 3000 rpm for 15 min at 4°C, before storing at −80°C until use. The middle of the ﬂap tissue (1×1 cm^2^ in size) from the proximal area of the vascular axis of the skin ﬂap was harvested, weighed, and diced into small blocks. One small piece of each tissue was fixed in 4% buffered paraformaldehyde, and the other parts were stored at −80°C. The flap tissues (about 100 mg) were homogenized in 1 mL normal saline at 4°C, centrifuged for 10 min at 10,000 g. The supernatant was collected, and the content of protein was detected.

### Tissue Edema Measurement

Tissue edema was reﬂected by water content. At 12 hours after the operation, an oven was used to dry the weighed new flap tissues for 24 h at 80℃. The percentage of water content was determined as follows:

Tissue water content (%)=(wet weight –dry weight)/wet weight×100%

### Determination of Nitrate and Nitrite Content

The concentrations of nitrate and nitrite in serum and tissue were detected by Total Nitric Oxide and Nitrate/Nitrite Parameter Assay Kit (PKGE001, R&D, USA) according to the manufacturer's instructions (Chang et al., 2019).

### Histological Staining

Flap specimens were fixed in 4% paraformaldehyde-buffered solution for 24h and processed with paraffin. The blocks were subsequently sectioned at 5 µm on the longitudinal plane and stained with hematoxylin and eosin (H&E) and Masson's Trichrome (MT).

The main histologic outcome was scored for inflammatory cell infiltration, collagen deposition, edema, hemorrhage, thrombosis, and hair follicle destruction as follows: (0) absent, (1) mild, (2) moderate, (3) severe (Ballestin et al., 2018). At least five microscopic areas were examined to score each specimen, and the total damage scores were calculated as the sum of the scores given for each criterion (Iseri et al., 2008).

### Immunohistochemical Staining

For immunohistochemical staining, tissues were fixed in 4% paraformaldehyde-buffered solution, incubated in 3% H_2_O_2_ for 20 min, blocked in 5% BSA for 2 h, and stained with primary antibodies against rat CD68 (1:1000 dilution, ab125212, Abcam, Cambridge, MA, USA) and MPO (1:1000 dilution, 14569T, Cell Signaling Technology, Danvers, MA, USA) for 8 h at 4°C. After that, biotinylated goat anti-rabbit IgG serum (Beijing Zhongshan Golden Bridge Biotechnology Co., Ltd., Beijing, China) was successively added to the sections for 3 h each at room temperature. Finally, the sections were stained with DAB Peroxidase Substrate Kit before imaging. Quantification was carried out in Image-Pro Plus version 6.0.

### Tunel Assay

Terminal deoxynucleotidyl transferase dUTP nick-end labeling (TUNEL) analysis was performed to measure the degree of apoptosis using the *in situ* Cell Death Detection kit (Roche Molecular Biochemicals, Mannheim, Germany) according to manufacturer's protocol. DAPI (4',6-diamidino-2-phenylindole) staining was performed to visualize nuclei after the TUNEL reaction. The apoptosis index was determined by counting TUNEL-positive nuclei in ten random fields per section and expressed as a percentage of the total nuclei.

### Evaluation of Antioxidant Defense and Oxidative Stress Biomarkers in Homogenate

The levels of catalase (CAT), glutathione peroxide (GSH-Px), superoxide dismutase (SOD), and Malondialdehyde (MDA) in tissue homogenate were evaluated using commercial kits (Nanjing Jiancheng Bioengineering Institute, Nanjing, China) according to the manufacturers' instructions (Liu et al., 2018).

### Inflammatory Cytokine Measurement

The concentrations of tumor necrosis factor alpha (TNF-α) and interleukin-6 (IL-6) in serum and tissue homogenate were detected using commercial ELISA kits (R&D Systems, Minneapolis, MN, USA) following the manufacturer's instructions. A microplate reader evaluated the optical density at 450 nm.

### Statistical Analysis

All data are presented as the mean ± SD. The statistical difference was evaluated by one-way analysis of variance (ANOVA) followed by Dunnett's test using IBM SPSS Statistics 22.0 software. p < 0.05 is considered as a statistically significant difference.

## Results

### Dietary Nitrate Attenuated IR-Induced Skin Flap Injury

**Figure 1:**
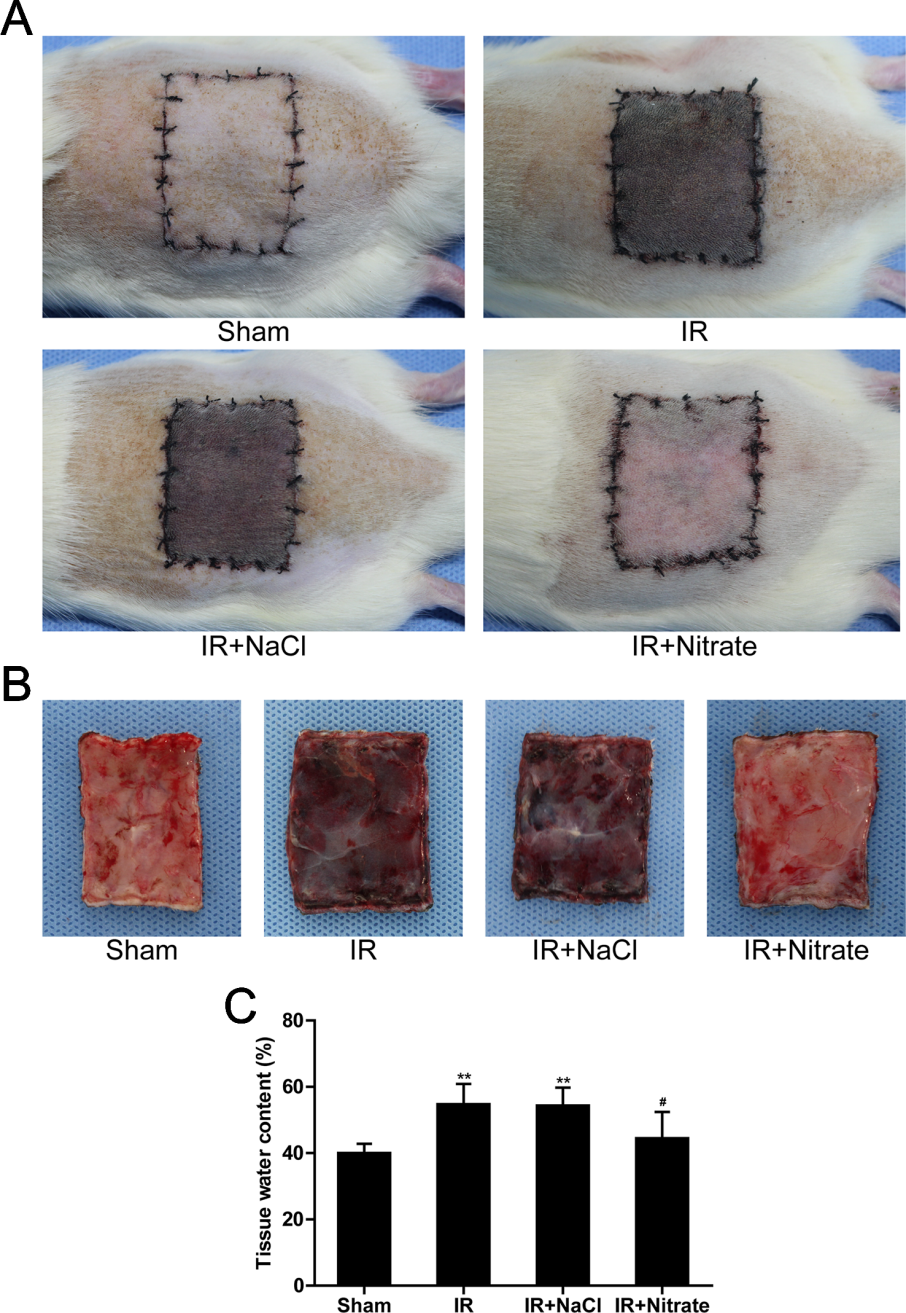
Dietary nitrate attenuated IR-induced skin flap injury. **(A, B)** Representative gross findings of the skin flap. **(C)** Percentages of tissue water content. Data are expressed as the mean ± SD, n = 6. **p < 0.01 versus Sham group; ^#^p < 0.05 versus IR group.

After IR, it was found that the flap was swollen and bruised, with venous blood stasis covering subcutaneous tissue. Nitrate pretreatment attenuated this IR-induced flap damage (**Figures 1A, B**). Compared with sham-operated rats, the tissue water content was obviously augmented in untreated IR rats, which was significantly decreased following nitrate supplement (**Figure 1C**).

### Dietary Nitrate Decreased Histological Lesions and Protected Cells From Apoptosis

**Figure 2:**
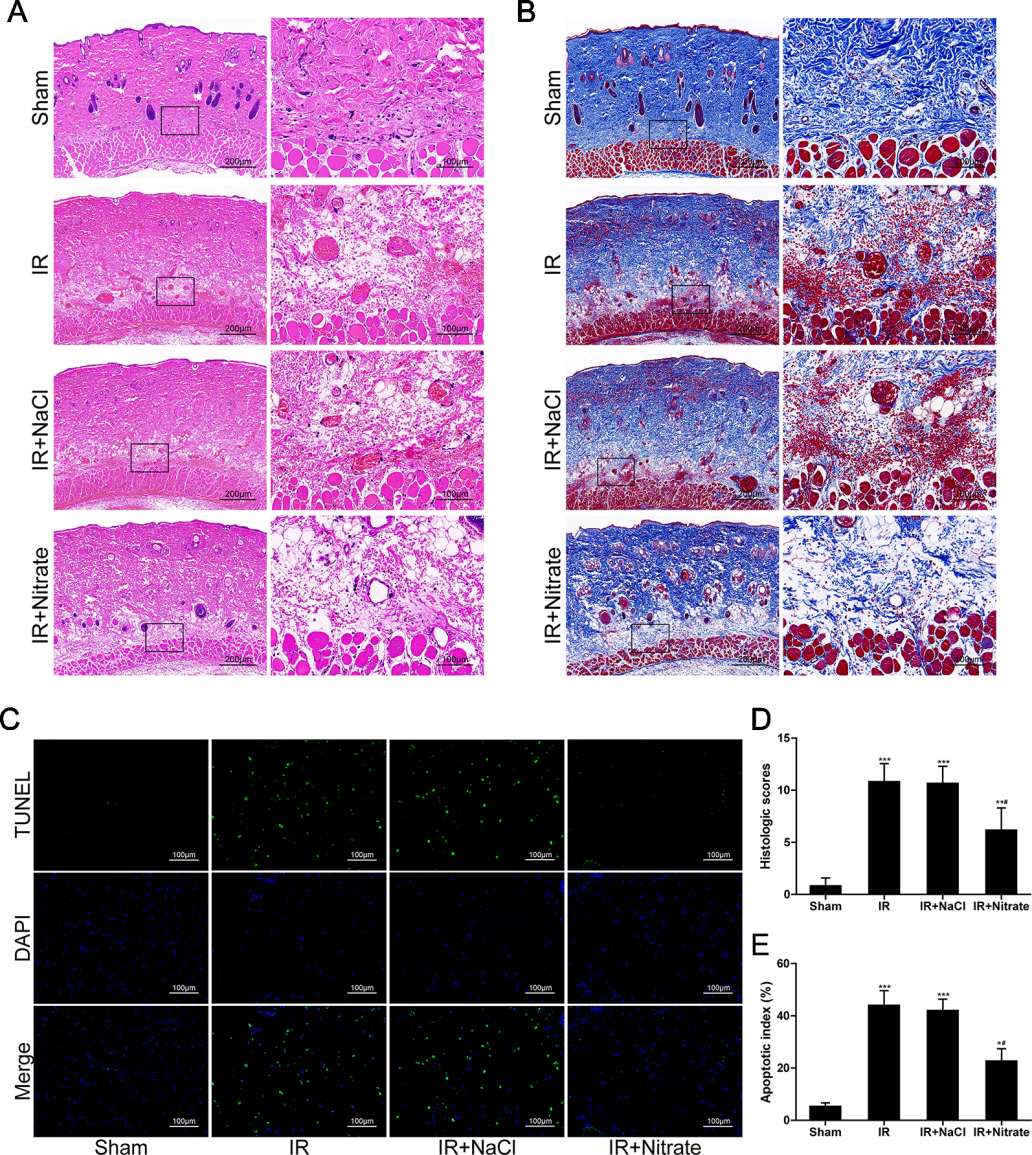
Dietary nitrate decreased histological lesions and protected cells from apoptosis. **(A**, **B)** H&E and Masson trichrome staining of the musculocutaneous flap. (Large image, scale bar = 200 µm; small image, scale bar = 100 µm). **(C)** Representative TUNEL-stained images of the skin flap. The apoptotic cells were detected by TUNEL (green), and the nuclei were detected by DAPI (blue). Scale bar = 100 µm. **(D)** Quantitative analysis of the histologic scores. **(E)** Apoptotic index of the flap. Data are presented as mean ± SD, n = 6. *p < 0.05, **p < 0.01, ***p < 0.001 versus Sham group; ^#^p < 0.05 versus IR group.

At 12 hours following ischemic insult, rats in the IR group showed distinct pathological changes, characterized by necrosis and inflammation in the ischemic flap. Particularly, massive cell infiltration and local hemorrhagic lesions were visible in perivascular connective tissue. These changes were significantly improved after dietary nitrate treatment (**Figures 2A, B**). Compared with sham-operated animals, flap IR induced a significant increase in the histology score measured from the flaps. The administration of nitrate significantly reduced the histology score compared with that of untreated rats (**Figure 2D**). TUNEL assay indicated that apoptosis was increased after 12 h of reperfusion but that this was reduced after nitrate treatment (**Figures 2C, E**).

### Dietary Nitrate Increased Nitrate and Nitrite Levels

**Figure 3:**
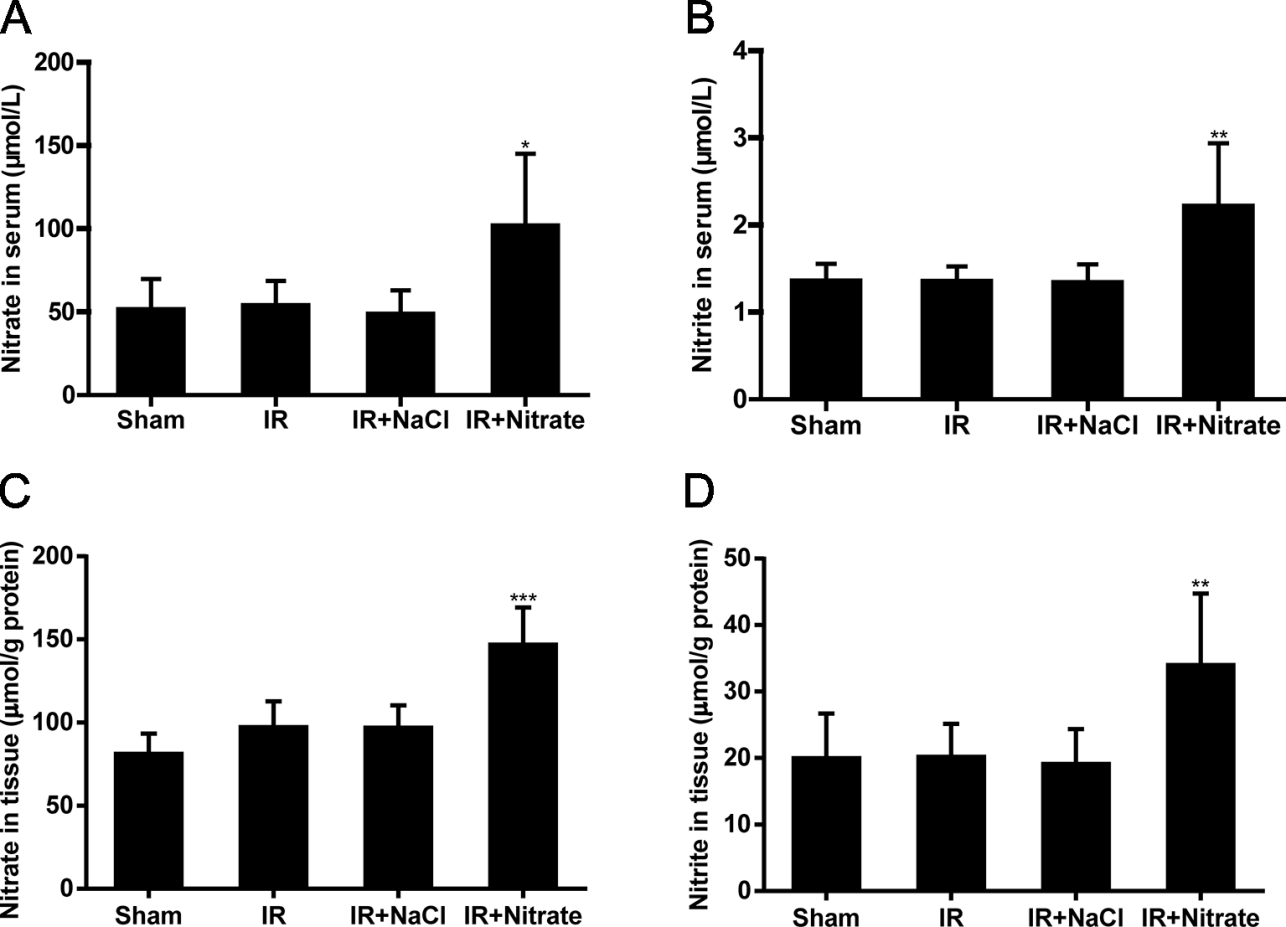
Dietary nitrate increased the levels of nitrate and nitrite. **(A**, **B)** Serum nitrate and nitrite levels after one-week nitrate feeding. **(C, D)** Flap tissue nitrate and nitrite levels after one-week nitrate feeding. Data are expressed as the mean ± SD, n = 6. *p < 0.05, **p < 0.01, ***p < 0.001 versus Sham group.

We further investigated the relationship between nitrate and nitrite through IR-induced flap injury in which the rats were fed a diet supplemented with nitrate. Daily intake of nitrate (5 mmol/L) increased the serum and flap tissue nitrate and nitrite levels in the IR rats compared with those in NaCl-intake IR rats (**Figure 3**).

### Dietary Nitrate Increases the Activity of Antioxidant Enzymes and Attenuates Oxidative Stress

**Figure 4:**
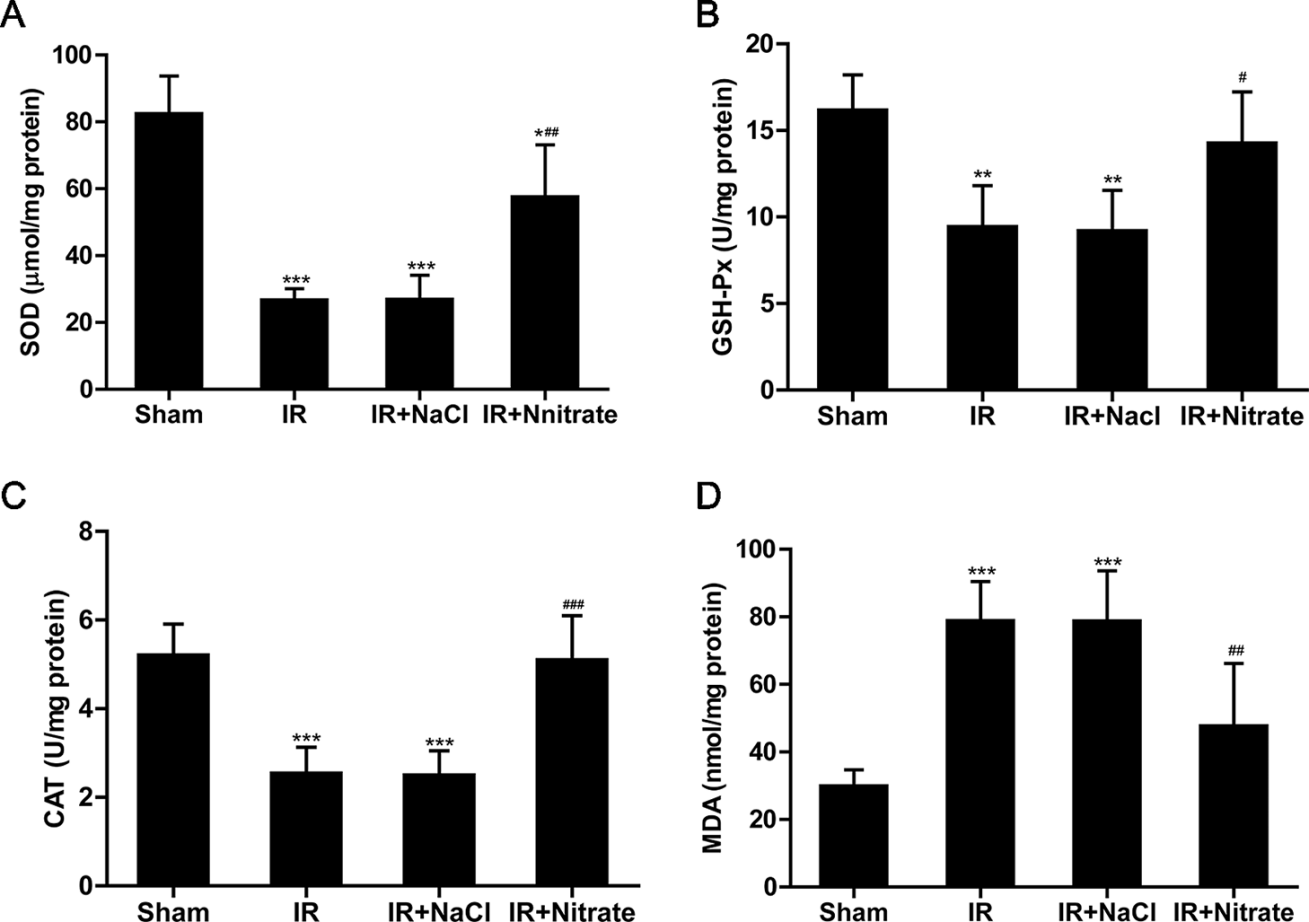
Dietary nitrate restored the activity of antioxidative enzymes and decreased lipid peroxidation after IR injury. Levels of superoxide dismutase (SOD) **(A)**; glutathione peroxidase (GSH-Px) **(B)**; catalase CAT **(C)** and malondialdehyde (MDA) **(D)** in the flaps following 12h of reperfusion. Data are expressed as the mean ± SD, n = 6. *p < 0.05, **p < 0.01, ***p < 0.001 versus Sham group; ^#^p < 0.05, ^##^p < 0.01, ^###^p < 0.001 versus IR group.

To further explore the potential mechanism of the beneficial effects of nitrate on IR injury, the activities of antioxidants were measured. As expected, IR caused a significant decrease in the activities of SOD, CAT, and GSH-Px compared with controls, and this was prevented by nitrate pretreatment. Additionally, after ischemia and 12 h reperfusion, the levels of MDA in tissue from nitrate-treated rats were decreased compared to non-treated groups (**Figure 4**).

### Dietary Nitrate Reduces IR-Associated Inflammatory Responses

**Figure 5:**
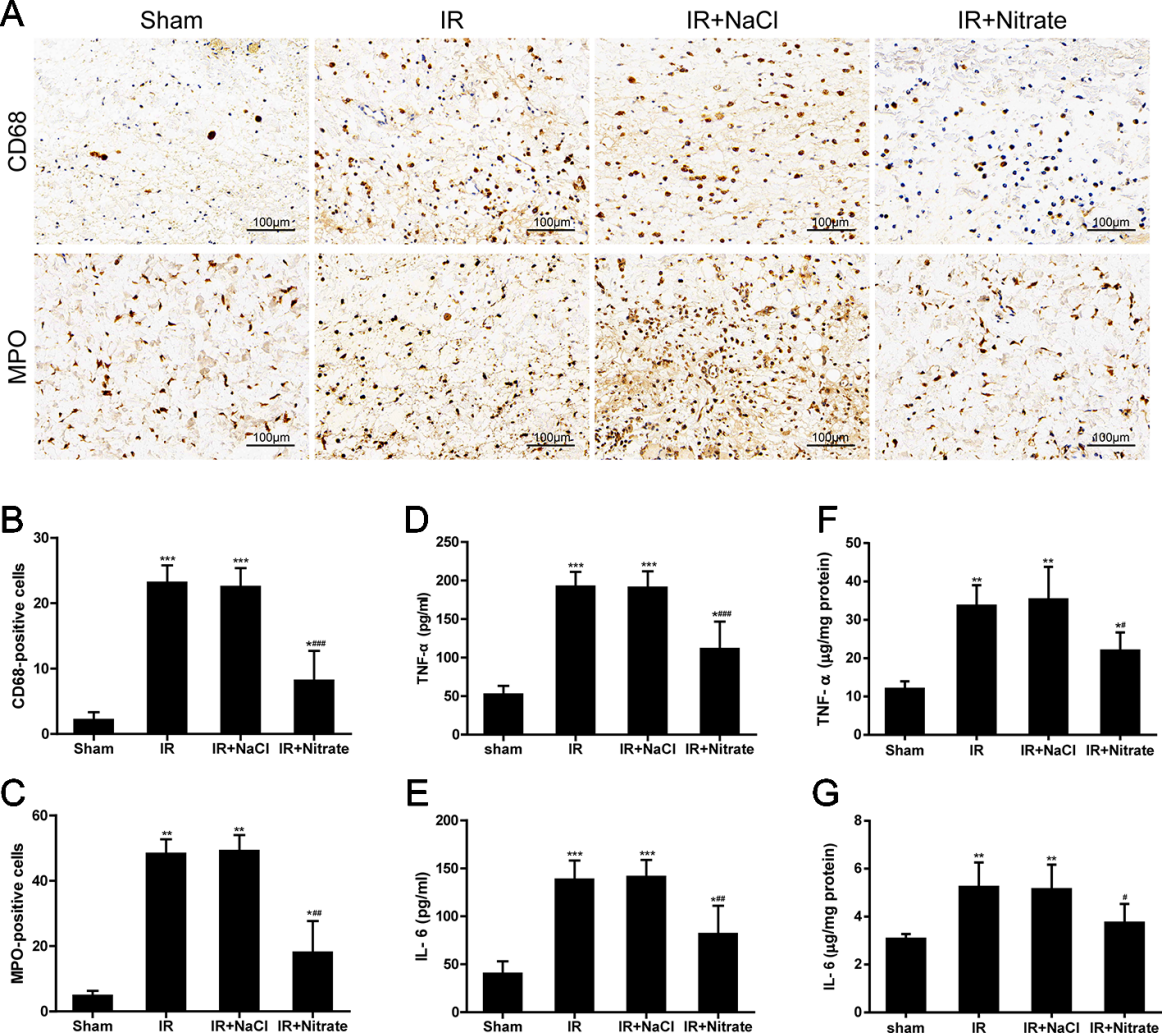
Dietary nitrate reduced inflammatory responses after IR injury. **(A)** Representative micrographs of immunohistochemistry for CD68 (macrophage) and MPO (leukocyte) of the skin flap. (Original magnification × 200, scale bar = 100 µm). **(B)** Numbers of CD68-positive cells. **(C)** Numbers of MPO-positive cells. **(D**, **E)** Levels of serum TNF-α and IL-6 in rats after flap IR injury. **(F**, **G)** Levels of flap issue TNF-α and IL-6 in rats after flap IR injury. Data are expressed as the mean ± SD, n = 6. *p < 0.05, **p < 0.01, ***p < 0.001 versus Sham group; ^#^p < 0.05, ^##^p < 0.01, ^###^p < 0.001 versus IR group.

To validate the local inflammatory changes in the skin flap following IR injury, macrophage and neutrophil infiltration were analyzed using CD68 and MPO immunostaining, and the cytokine levels were measured in ischemia flaps following 12 h of reperfusion. There was substantial infiltration of macrophages and neutrophil granulocytes in the tissue of the ischemic flap (**Figures 5A–C**). Compared to non-IR controls, the ischemic flap from non-treated and NaCl rats had significantly higher TNF-α and IL-6 levels in serum and flap tissue. In nitrate-treated rats, the levels of TNF-α and IL-6 were both significantly lower (**Figures 5D–G**).

## Discussion

The current study investigated the effects of intake of a nitrate supplementation diet on the skin flap following IR injury. We found that dietary nitrate protects against IR-induced tissue apoptosis by enhancing antioxidant activity and reducing the inflammatory response. This is the first demonstration of the role of dietary nitrate in protecting the skin flap against IR injury.

Many researchers have set 6-10 hours as the ischemic duration in their experiments. Significant tissue damage, including endothelial dysfunction, vasoconstriction, and thrombosis in microcirculation, has been seen within this timeframe (Cetin et al., 2001; Wang et al., 2009; Muneuchi et al., 2013; Xiao, 2016). Hatoko found that the levels of apoptotic cells were significantly elevated in rats that had undergone 12 or more hours of reperfusion (Hatoko et al., 2001). Moreover, many researchers have established their reperfusion periods at 12 hours or more time in their IR injury rat models (Cordeiro et al., 2000; Lee et al., 2017). In our study, we studied this process using the model of ischemia-reperfusion flaps, which experience predictable and significant amounts of flap tissue necrosis. Thus, it was reasonable to design experiments including 10 h of ischemia and 12 h of reperfusion.

The harmful role of inorganic nitrate and its downstream products has been shown to be due to its bioconversion to carcinogenic nitrosamines (Tannenbaum and Correa, 1985; Bedale et al., 2016). However, it is clear that nitrate can be continuously metabolized to NO and other bioactive nitrogen oxides (Lundberg et al., 2004; Lundberg et al., 2008a; Hezel et al., 2015). Particularly, leafy vegetables contain high amounts of anion nitrate. Inorganic nitrate from dietary sources can be reduced to nitrite by commensal bacteria in the mouth and gastrointestinal tract (Lundberg and Govoni, 2004). Nitrite is then converted to NO and nitrogen oxides in blood and tissues *via* multiple pathways (Bryan, 2006). Many studies have demonstrated the benefits of dietary nitrate, which may boost the nitrate-nitrite-NO pathway in the body (Lundberg et al., 2011a; Sindler et al., 2014b; Husmann et al., 2019). This distinct alternative pathway for NO generation is independent of NO synthase (NOS). Therefore, the nitrate-nitrite-NO pathway would be of particular importance in the ischemic and hypoxic processes while NOS function is suppressed (Hezel et al., 2016a).

Dietary nitrate is an effective way of delivering nitrite systemically. With a half-life of 6 hours, it continuously generates nitrite and bioactive nitrogen oxides (Pannala et al., 2003; Butler and Feelisch, 2008). Dietary nitrate needs to be constantly ingested in order for the individual to benefit from the beneficial effects and other biological functions of NO. The doses of administration for nitrate used in this study were selected based on previous studies regarding liver, heart, and kidney IR injury (Hendgen-Cotta et al., 2012). Our previous research has also demonstrated that dietary 5 mmol/L nitrate can effectively enhance blood flow following ischemia-induced injury (Cui et al., 2019). Thus, 5 mmol/L nitrate was administered to the treated group in this study.

The bioactivation of nitrate is related to the initial reduction to NO, which is a key regulator during health maintenance and disease (Lundberg et al., 2008b). NO can induce vasodilation and the inhibition of oxidative stress, leukocyte chemotaxis, platelet aggregation, and apoptosis (Ignarro et al., 1987; Ma et al., 1993; Li et al., 1999). Moreover, NO has key roles during IR injury (Webb et al., 2004; Peralta et al., 2013). Reduced NO bioavailability due to increased oxidative stress and inflammation has been considered to a central element contributing to flap IR injury. Thus, the balance of NO bioavailability may have implications for a novel strategy against skin flap IR injury. In the present study, we demonstrated that oral administration of nitrate significantly increases concentrations of nitrate and nitrite, which accounts for the pivotal role of nitrate in protection against IR injury.

Oxidative stress is one of the common factors resulting from IR injury. Generation of reactive oxygen species leads to excessive production of cytotoxic metabolite, which enhances injury membrane lipoproteins, protein oxidation, and oxidative DNA damage (Noiri et al., 2001). Also, NO has been shown to attenuate the burden of oxygen free radicals through regulating mitochondrial complex I (Totzeck et al., 2017). In this study, the induction of IR injury in skin flap significantly decreased antioxidant defense systems and enhanced lipid peroxidation in skin flap. The results suggested that nitrate as a biological reservoir of NO exerts protective effects during IR injury through reducing oxidative stress. This is in line with previous studies that showed that dietary nitrate supplementation can exert protective effects against endothelial dysfunction, metabolic diseases, and myocardial infarction by restoring NO bioavailability and targeting oxidative stress (Carlstrom et al., 2011; Hezel et al., 2016b).

In the meantime, the release of inflammatory mediators is another pivotal contributor to cell death during IR injury. After reperfusion, inflammatory cell infiltration in injured tissues is responsible for the production of inflammatory factors, including TNF-α and IL-6, which subsequently trigger a feedback loop in inflammatory cascades, leading to tissue dysfunction and damage (Aboutaleb et al., 2019). Moreover, inflammatory cytokines can further activate the immune/inflammatory response and result in the extension of injury (Zhao et al., 2013). Consequently, cellular apoptosis and tissue necrosis are induced. Our findings showed that expression levels of inflammatory cytokines were markedly increased in rats subjected to flap IR injury and were reversed by nitrate. Also, the number of CD68-positive macrophages and MPO-neutrophils were significantly increased in the ischemic flap following 12h of reperfusion and were reversed by nitrate. Therefore, dietary nitrate improved IR injury along with suppressing the inflammatory response.

Species differences should be taken into consideration when comparing human and animal serum nitrate concentrations. The concentration of nitrate we used does not reflect the concentration in human serum. In healthy subjects, normal serum levels of nitrate are in the 20-40 µmol/L range, and nitrite levels are in the 50-300 nmol/L range (Pannala et al., 2003; Lundberg et al., 2008a). Most of the potential favorable effects of treatment with nitrate observed in animals also occur in humans. The amount of nitrate we used in this study is possible to achieve by the daily consumption of nitrate-rich vegetables.

In conclusion, intervention with nitrate could effectively protect against IR-induced skin flap injury, an effect that is coupled to the antioxidative and anti-inflammatory properties of nitrate. These findings support the argument that dietary nitrate is a potential preventive and therapeutic strategy for improving IR-induced skin flap injury.

## Data Availability Statement

The raw data supporting the conclusions of this article will be made available by the authors, without undue reservation, to any qualified researcher.

## Ethics Statement

Our study was provided with ethical approval by the local animal welfare committee of the Affiliated Hospital of Qingdao University (Protocol No. AHQU20190107A).

## Author Contributions

HC designed and performed all the experiments. YF, CS, LB, and RY participated in the animal study. BP and MJ supervised the entire study.

## Funding

This work was supported by the Shandong Provincial Natural Science Foundation (ZR2017BH034).

## Conflict of Interest

The authors declare that the research was conducted in the absence of any commercial or financial relationships that could be construed as a potential conflict of interest.
