# Supplementary material for: Dietary Nitrate Protects Against Skin Flap Ischemia-Reperfusion Injury in Rats via Modulation of Antioxidative Action and Reduction of Inflammatory Responses
Source: Front Pharmacol. 2020 Jan 22;10:1605. doi: 10.3389/fphar.2019.01605 (PMC6987438; doi:10.3389/fphar.2019.01605)
Supplement: Supplementary file 1 [file Image_1.pdf]

Supplementary Figure

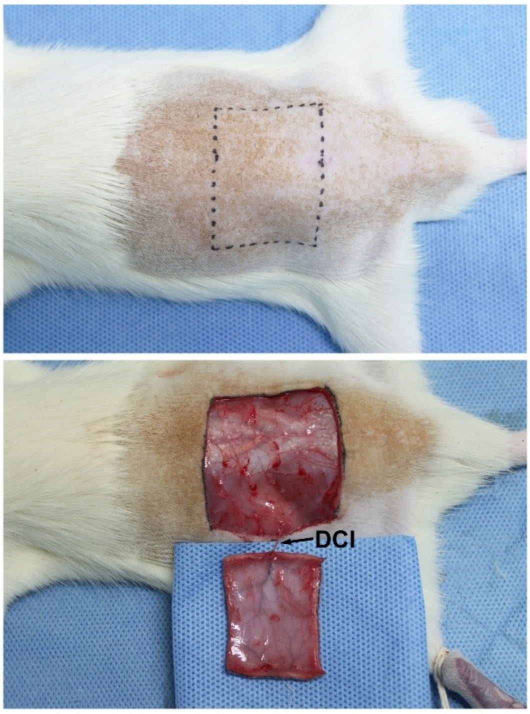

Supplemental Figure 1. Design of the flap based on the left deep circumflex iliac vessels (DCI). A dorsal rectangular flap (3×4 cm in size) was elevated, retaining only the DCI as the vascular pedicle. DCI, deep circumflex iliac vessel.
